# Draft Genome Sequence of *Mycobacterium triplex* DSM 44626

**DOI:** 10.1128/genomeA.00499-14

**Published:** 2014-05-29

**Authors:** Mohamed Sassi, Olivier Croce, Catherine Robert, Didier Raoult, Michel Drancourt

**Affiliations:** Aix Marseille Université, URMITE, Marseille, France

## Abstract

We announce the draft genome sequence of *Mycobacterium triplex* strain DSM 44626, a nontuberculosis species responsible for opportunistic infections. The genome described here is composed of 6,382,840 bp, with a G+C content of 66.57%, and contains 5,988 protein-coding genes and 81 RNA genes.

## GENOME ANNOUNCEMENT

*Mycobacterium triplex* was described on the basis of a unique mycolic-acid pattern and a distinctive 16S rRNA gene hypervariable region (1). Phylogenetic analysis confirmed close relationships with *Mycobacterium simiae*, *Mycobacterium genavense*, *Mycobacterium lentiflavum*, and *Mycobacterium sherrisii* (2, 3). *M. triplex* has been initially isolated from sputum (4), lymph nodes (5), and cerebrospinal fluid specimens (1, 6). Only six cases of infection have been reported in immunocompromised patients, including HIV-infected patients (1) and a liver transplant patient (7).

We analyzed the whole-genome sequence of *M. triplex* to facilitate the elucidation of its relationships within the *M. simiae* complex and the design of tools for its advanced detection and identification.

Genomic DNA was isolated from *M. triplex* strain DSMZ 44626 grown in MGIT Middlebrook liquid culture (Becton Dickinson, Sparks, MD) at 37°C. DNA was sequenced using three high-throughput next-generation sequencing (NGS) technologies: Roche 454 (Roche Diagnostics Corporation, Indianapolis, IN) (8), SOLiD version 4 (Life Technologies, Carlsbad, CA), and MiSeq Illumina (Illumina, Inc., San Diego, CA). Two Roche 454 libraries, a 3.6-kb paired-end and a 1.6-kb shotgun XL+, were constructed, loaded on a picotiter (PTP) plate, and sequenced with the Roche-GS FLX Titanium sequencing kit XLR70. The two runs yielded 90.13 Mb with 265,087 passed filters and an average 348-bp length. The bar-coded paired-end SOLiD library generated 1,338,576 reads of 50- × 35-bp-length reads. Finally, a paired-end Nextera library, fragmented at 942 bp and sequenced on MiSeq in 2 × 151 bp, yielded 269,610 reads with an indexing of 2.15% on the flow cell.

The reads that issued from these three sequencing technologies were first assembled separately. The 454 reads were assembled into contigs and scaffolds using Newbler version 2.8 (Roche). Illumina reads, trimmed using Trimmomatic (9), were assembled using the Spades software (10, 11). Contigs obtained were combined by using SSpace (12) and Opera v. 1.2 (13) software; GapFiller v. 1.10 (14) helped to reduce the set. Some manual refinements using CLC Genomics v. 5 software (CLC bio, Aarhus, Denmark) improved the genome.

These analyses yielded four scaffolds of 21 contigs containing 6,379,625 bp and an estimated size, including gaps, of 6,382,840 bp, for a 66.57% G+C content. Noncoding genes and miscellaneous features were predicted using RNAmmer (15), ARAGORN (16), Rfam (17), and PFAM (18). Open reading frames (ORFs) were predicted using Prodigal (19) and functional annotation was achieved using BLASTP against the GenBank database (20) and the Clusters of Orthologous Groups (COGs) database (21, 22). The genome was shown to encode at least 81 predicted RNAs, including 3 rRNAs in a single operon, 58 tRNAs, 1 transfer-messenger RNA (tmRNA), and 19 miscellaneous RNAs. Also, 5,988 genes represented a coding capacity of 5,867,145 bp for a 91.9% coding percentage. Among these genes, 765 (12.77%) were found to encode putative proteins and 1.011 (16.88%) were assigned hypothetical proteins. Moreover, 5,940 genes matched a least one sequence in the COGs database with BLASTP default parameters.

### Nucleotide sequence accession numbers.

The *M. triplex* strain DSM 44626 genome sequence has been deposited at ENA under the accession numbers CCAU010000001 to CCAU010000021.
